# Regulatory microRNAs and vascular cognitive impairment and dementia

**DOI:** 10.1111/cns.13472

**Published:** 2020-11-30

**Authors:** Jing Zhang, Ping Sun, Chao Zhou, Xuejing Zhang, Feifei Ma, Yang Xu, Milton H. Hamblin, Ke‐Jie Yin

**Affiliations:** ^1^ Department of Neurology Pittsburgh Institute of Brain Disorders & Recovery University of Pittsburgh School of Medicine Pittsburgh PA USA; ^2^ Department of Pharmacology Tulane University School of Medicine New Orleans LA USA; ^3^ Geriatric Research Education and Clinical Center Veterans Affairs Pittsburgh Healthcare System Pittsburgh PA USA

**Keywords:** aging, dementia, microRNAs, regulatory mechanisms, vascular cognitive impairment

## Abstract

Vascular cognitive impairment and dementia (VCID) is defined as a progressive dementia disease related to cerebrovascular injury and often occurs in aged populations. Despite decades of research, effective treatment for VCID is still absent. The pathological processes of VCID are mediated by the molecular mechanisms that are partly modulated at the post‐transcriptional level. As small endogenous non‐coding RNAs, microRNAs (miRs) can regulate target gene expression through post‐transcriptional gene silencing. miRs have been reported to play an important role in the pathology of VCID and have recently been suggested as potential novel pharmacological targets for the development of new diagnosis and treatment strategies in VCID. In this review, we summarize the current understanding of VCID, the possible role of miRs in the regulation of VCID and attempt to envision future therapeutic strategies. Since manipulation of miR levels by either pharmacological or genetic approaches has shown therapeutic effects in experimental VCID models, we also emphasize the potential therapeutic value of miRs in clinical settings.

## Graphical abstract

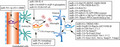

## 1 INTRODUCTION

Vascular cognitive impairment and dementia (VCID) is currently the second most common type of dementia just after Alzheimer's disease (AD).^1^ In 2015, approximately 47.5 million people were affected by dementia, which is expected to increase to 75.6 million by 2030. The annual global social cost for VCID and vascular dementia (VaD) is $604 billion, accounting for 1.0% of the global gross domestic product.^2^

Vascular cognitive impairment and dementia occurs when cerebral blood flow is compromised. It is a comprehensive brain disorder that comprises mild cognitive impairment (MCI), VaD, and mixed dementia, such as mixed vascular and AD‐type cognitive impairment.^3^ VCID presents a significant decline in cognitive function due to cerebral vascular damage, including clinical stroke, asymptomatic infarcts and microinfarcts, leukoaraiosis, cerebral amyloid angiopathy (CAA),^4^ transient ischemic attack (TIA), and micro hemorrhage.^5^ Diagnosis is further defined according to whether there is a causal relationship between cognitive impairment syndrome and vascular disease.^6^, ^7^

Up to now, treatment for VCID is still limited to relief and therapy of symptoms.^8^ For example, Donepezil was found to enhance the cognitive ability of VaD patients.^3^ Administration of Galantamine is beneficial for patients with mixed AD and VaD.^9^

Non‐coding RNAs, especially miRs, are one of the many biological factors that cause functional changes during VCID. Because of the redundancy of targets, miRs can target multiple signal pathways; one miR can target multiple messenger RNAs (mRNAs), whereas numerous miRs can act on one mRNA at the same time.^10^ VCID can trigger altered miR expression in the blood and brain of rodents and humans.^11^, ^12^, ^13^, ^14^ Besides, miRs can be regulated by external agents to improve symptoms caused by VCID.^15^, ^16^, ^17^, ^18^

In this review, we summarize the current advances on the pathogenesis and treatment of VCID, with a focus on the possible role of miRs in disease regulation and attempt to explore future therapeutic strategies.

## 2 OVERVIEW OF VASCULAR COGNITIVE IMPAIRMENT AND DEMENTIA (VCID)

Vascular cognitive impairment and dementia includes any degree of cognitive impairment resulting from vascular brain pathology, from MCI to dementia, regardless of its specific mechanism.^19^

### 2.1 White matter injury in VCID

Given that blood flow in white matter (WM) is supplied by long, penetrating arterioles that lack anastomotic branches, WM is more susceptible to reduced CBF.^20^, ^21^ WM is composed of neuronal axons, the surrounding myelin sheath, and glial cells such as astrocytes, oligodendrocytes, pericytes, and microglia.^22^ Myelinated WM tracts are responsible for long‐range connectivity through axonal transport, and their lesions can lead to neuronal circuits processing speed deficits and corticocortical disconnections.^23^ The pathological changes in WM can predict VCID according to neuropathology guidelines, and WM injury is a significant contributor to dementia.^24^, ^25^, ^26^

In animal models of VCID, permanent occlusion of the common carotid arteries (CCAs) is the most frequently used large vessel occlusion model that leads to BBB disruption and significant WM impairment.^27^ This model shares several common pathological consequences with small vessel disease, including microinfarcts and WM changes.^28^, ^29^ WM lesions and rarefaction mainly occur in the corpus callosum with remarkable myelin loss, axonal damage, microglia, and astrocyte activation, without causing neuronal damage.^30^, ^31^, ^32^, ^33^ In the model of small vessel occlusion, microglia/macrophage polarization was also found strongly linked to VCID.^34^ Except for the rodent models, the accumulation of myelin defects such as myelination, swelling, and complete axonal degeneration has also been found to be correlated with cognitive decline in rhesus monkeys.^35^ Besides, aging exacerbates the degeneration of WM, and the consequences of chronic cerebral hypoperfusion (CCH) are more severe in aged animals.^36^

### 2.2 Gray matter injury in VCID

The neurovascular unit consists of neurons, glia, perivascular, and vascular cells. Together, they maintain the normal physiological function of neurons, repair damaged neurons, and play an essential role in keeping the homeostasis of the cerebral microenvironment.^37^ The pathogenesis of VCID mainly appears in the neurovascular unit. Among these neurovascular components, the neuron is the basic structural and functional unit of the nervous system. Global neuronal loss that is produced by persistent cerebral hypoperfusion in specific brain regions, such as the hippocampus, can lead to severe learning and memory impairment.^38^

Neurons are electrically active cells that require a continuous supply of oxygen and glucose to produce a tremendous amount of energy, which is needed to maintain membrane potential. Therefore, neurons are vulnerable to ischemic injury. Different from stroke, which is induced by a sudden and complete disruption of blood supply to different regions of the brain, non‐stroke causes of VCID is often caused by a moderate but sustained decrease in blood supply by CBF. The mild but continuous reduction of blood supply could cause a reduction in oxygen and glucose supply to the brain, leading to cell death, memory impairment, and dementia.^39^

### 2.3 Molecular mechanism of VCID

**Figure 1:**
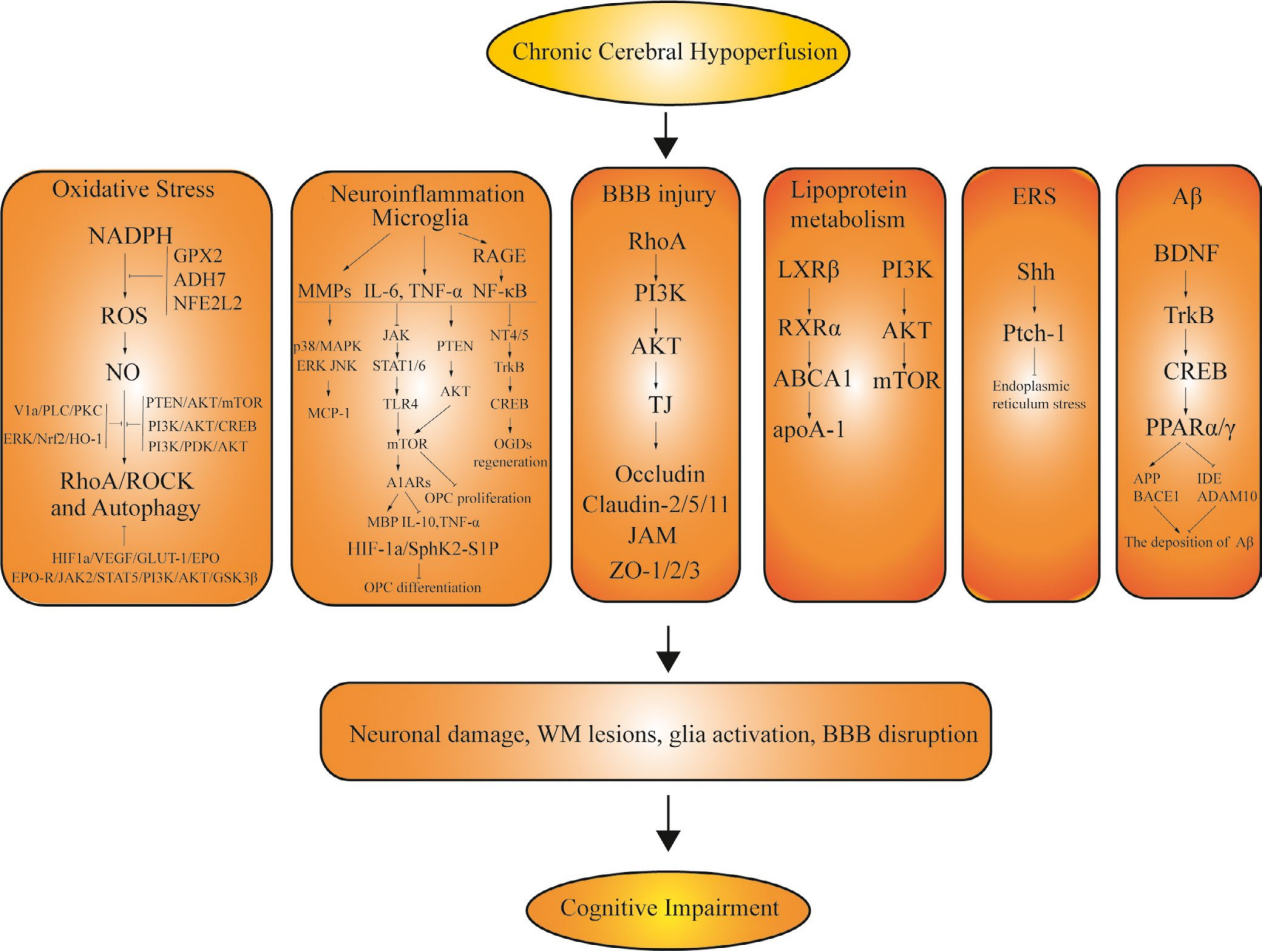
Potential molecular mechanisms in VCID. CCH can cause a cascade of pathological changes: neuronal damage, WM lesions, glial activation, and BBB disruption, resulting in cognitive impairment and dementia in experimental studies. The following cellular hemostasis abnormalities mainly contribute to the above pathological changes in VCID: oxidative stress, neuroinflammation, BBB disruption, abnormal lipoprotein metabolism, endoplasmic reticulum stress, and the deposition of Aβ

Although animal models cannot entirely present the complex clinical symptoms of VCID in humans, they aid in understanding the molecular changes in the brain during cerebrovascular injury to a certain extent, which eventually leads to cognitive impairment of VCID.^40^ Several mechanisms can be used to generalize the pathological causes of the VCID at the molecular level (Figure 1).

#### 2.3.1 Oxidative stress

The molecular mechanisms of oxidative stress‐induced cognitive impairments can be studied by using animal models, such as bilateral carotid artery occlusion (BCAO) rat models. In the BCAO model, oxidative stress is characterized by an increase in ROS production,^41^ which is one of the triggers leading to cardiovascular pathophysiology and neurodegeneration.^42^ The accumulation of ROS and the decrease in antioxidant enzymes may directly affect the synaptic activity and excitatory transmission of neurons, leading to cognitive impairment.^43^ The enzyme that produces excessive ROS production during VCID is nicotinamide adenine dinucleotide phosphate (NADPH). ^43^ ROS produced by NADPH co‐enzymes are the critical contributors to cerebrovascular dysregulation and might lead to cognitive impairment through cell dysfunction and cell death.^43^ Inhibition of NADPH oxidase activity reduced the cognitive impairment induced by BCAO models in rodents.^44^ Similar alterations are also observed in VCID patients. Several studies have reported that the level of antioxidant enzymes in blood samples of VaD patients is decreased.^45^, ^46^

There are many signal pathways involved in the regulation of oxidative stress in VCID. Some researchers reported that activation of the PI3K/PDK1/AKT pathway could inhibit the apoptosis of neurons in VCID through antioxidative stress effects.^47^ The activated ERK‐Nrf2‐HO‐1 signaling pathway is also related to the antioxidant protection in response to hypoperfusion injury.^48^ L‐carnitine is an antioxidant agent, which can regulate PTEN/the mammalian target of rapamycin (mTOR) signaling pathway in the rat CCH model, thereby enhancing axonal plasticity and oligodendrocyte expression.^49^

#### 2.3.2 Neuroinflammation

Neuroinflammation is closely involved in the pathophysiology of VCID.^50^ Inflammatory‐related microglia causes cognitive impairment by activating receptor for advanced glycation end products (RAGE), which is present on both microglia and neurons. RAGE can further stimulate the expression of nuclear factor kappa B (NF‐κB), a transcription factor that regulates the expression of several pro‐inflammatory genes.^51^ Besides, microglia releases cytokines, such as interleukin (IL) and tumor necrosis factor‐α (TNF‐α), that play essential roles in the pathogenesis of dementia.^52^, ^53^ Increased IL‐6 is associated with VaD in patients.^54^ Accordingly, the serum IL‐6 level of VaD patients also increased significantly.^55^

#### 2.3.3 BBB integrity and injury in VCID

Vascular cognitive impairment and dementia is a cerebrovascular injury‐related disease associated with BBB disruption. Endothelial tight junction (TJ) proteins are major components of the BBB and are responsible for sealing gaps between adjacent endothelial cells.^56^ Altered distribution or loss of TJ proteins is frequently seen in ischemic‐induced cerebral microvessel injuries, resulting in compromised BBB integrity and dementia.^57^ TJ proteins include transmembrane proteins, cytoplasmic attachment proteins, and cytoskeletal proteins.^58^ Transmembrane proteins include three complete membrane proteins, occludins, claudins, and junctional adhesive molecules (JAMs). Cytoplasmic attachment proteins, which are also named as closed small loop proteins, contain ZO‐1, ZO‐2, and ZO‐3. Prolonged hypoperfusion in white matter and gray matter eventually leads to TJ disruption and BBB leakage, which come into appearance before cognitive impairments.^59^ As the most common model in VCID studies, the BCAS model shows BBB leakage not only in the corpus callosum and external capsule but also in the gray matter.^60^ A meta‐analysis from 31 studies counted 1953 individuals of normal aging or cerebral microvascular disease. In 693 healthy human, increasing age was associated with the increase in BBB permeability. BBB permeability was increased further in 510 patients with either VCID or AD presented compared with 547 aged‐matched controls.^59^, ^61^ In the post‐mortem brains of VCID patients, there are higher levels of claudin‐2, claudin‐5, and claudin‐11.^62^ Besides, claudin‐1 genetic polymorphisms were found to be highly associated with VCID.^63^ Besides TJ disruption and subsequent paracellular leakage, the reverse transcytosis across BBB is also involved in the clearance of Aβ from brain.^64^, ^65^ Failure or reduction in Aβ brain clearance through endothelial transcytosis may lead to the accumulation of Aβ in brain and finally result in dementia.^66^

#### 2.3.4 Other mechanisms

In recent years, cumulative evidences have shown that the pathogenesis of VCID is closely related to the destruction of cholesterol homeostasis and lipoprotein disturbances.^67^, ^68^ Changes in cholesterol homeostasis lead to abnormal cholesterol uptake from plasma to brain.^69^ Apolipoproteins (such as ApoA, ApoE) and the cholesterol efflux transporter, ABCA1 (ATP‐binding cassette transporter A1), are involved in the cholesterol conversion between astrocytes and neurons in the brain.^70^ Liver X receptor‐β/retinoid X receptor‐α (RXR‐α)/ABCA1 signaling cascade plays a vital role in lipoprotein metabolism.^71^, ^72^, ^73^ ApoA1 and cholesterol, the downstream mediators of this signaling pathway, may provide a protective role in cerebral hypoperfusion.^70^ Fatty acid amide hydrolase (FAAH) inhibitor UBR597 blocks the PI3K‐AKT‐mTOR pathway and autophagy to attenuate CCH‐induced neuronal damage and improve cognitive function.^74^ Curcumin can reduce the discharge of excess cholesterol and prevent further brain injury by activating the LXR/RXR‐ABCA1/apoA‐1 pathway.^75^

Endoplasmic reticulum stress (ERS) is a process by which unfolded/misfolded proteins accumulate in the ER after an ER homeostasis disorder.^76^ Specific stress conditions such as hypoxia, nutrient deprivation, calcium consumption, and hyperglycemia can trigger ERS.^77^ Sustained ERS eventually leads to cell apoptosis. N‐Butylphthalide (NBP) can reduce ERS and treat VCID by activating the Shh/Ptch1 pathway in the hippocampus.^78^

It is estimated that 40% of AD patients also have some forms of VCID.^79^, ^80^, ^81^, ^82^ The most common hypothesis for the progression of AD is the amyloid cascade hypothesis, stating that beta‐amyloid (Aβ) aggregation leads to hyperphosphorylation of tau and tangle formation, which then leads to neurodegeneration.^83^, ^84^ CCH can induce the deposition of Aβ in the hippocampus,^85^ along with neuronal morphological damage and cognitive deficits.^86^, ^87^, ^88^, ^89^ Icariin can downregulate the level of insoluble Aβ fragments in the hippocampus by decreasing the expression levels of Aβ‐protein precursor (APP) and β‐site APP‐cleaving enzyme 1 (BACE1), while increasing the expression levels of insulin‐degrading enzyme (IDE) and ADAM metallopeptidase domain 10 (ADAM10). The effect of Icariin on Aβ reduction is mediated by upregulation of peroxisome proliferator‐activated receptor α (PPARα) and γ (PPARγ), and the activation of BDNF/TrkB/CREB signaling pathway.^90^

## 3 MICRORNAS AND VCID

miRs function as a novel class of small non‐coding RNAs (~21‐25 nt) that negatively regulate gene expression.^91^, ^92^ By hybridizing to the 3’‐untranslated regions (3’‐UTR) of one or more mRNAs, miRs negatively regulate gene expression.^91^, ^92^ miRs involve almost all cellular processes, including cell proliferation, differentiation, metabolism, apoptosis, and immune responses in various pathophysiological conditions.^93^

### 3.1 The biomarker functions of miRs in VCID

miRs are very stable in various biofluids, including blood, plasma, CSF, and saliva,^93^, ^94^, ^95^ and thus, circulating miRs can serve as potentially informative biomarkers for a range of neurological diseases (Table 1). By performing plasma miR profiling in small‐vessel VaD patients and also in age‐ and sex‐matched healthy controls, Prabhakar et al demonstrated that among the 44 differentially expressed miRs, miR‐409‐3p decreased more than 4‐fold whereas miR‐451a, miR‐486‐5p, and miR‐502‐3p increased more than 3.6‐fold compared with healthy controls.^96^ A validation study further suggested these miRs as potential biomarkers for identifying small‐vessel VaD.^96^ Sheinerman et al also found that two sets of circulating brain‐enriched miRs, the miR‐132 family (miR‐128, miR‐132, and miR‐874) and the miR‐134 family (miR‐134, miR‐323‐3p, and miR‐382), were significantly different in MCI patients from age‐matched controls with very high sensitivity and specificity.^97^ In a recent study, Marchegiani et al also reported that compared with both healthy controls and dementia patients, the level of miR‐222 was significantly increased in VaD patients, suggesting miRs are novel and promising biomarkers to diagnose VaD.^98^

**Table 1:** MicroRNAs as potential biomarker in VCID

MicroRNAs	Biomarkers	Sensitive (%)	Specificity (%)	Reference
miR‐409‐3p	Diagnosis	76	89	Langa et al^81^
miR‐451a	Diagnosis	70	75	Langa et al^81^
miR‐486‐5p	Diagnosis	75	83	Langa et al^81^
miR‐502‐3p	Diagnosis	75	89	Langa et al^81^
miR‐128	Diagnosis	84	96	Sheinerman et al^97^
miR‐132	Diagnosis	88	93	Sheinerman et al^97^
miR‐874	Diagnosis	94	96	Sheinerman et al^97^
miR‐134	Diagnosis	86	82	Sheinerman et al^97^
miR‐323‐3p	Diagnosis	88	80	Sheinerman et al^97^
miR‐382	Diagnosis	76	90	Sheinerman et al^97^
miR‐222	Diagnosis	N/A	N/A	Marchegiani et al^98^

### 3.2 The role of microRNAs in the pathogenesis of VCID

Apart from the biomarker functions of miRs, accumulating evidence also revealed the critical role of miRs in the pathogenesis of VCID in animal models (Table 2). miR‐195 was the first systematically investigated miR in CCH‐induced cognitive impairment. Ai et al demonstrated that miR‐195 repressed amyloidogenesis via regulating the expression of APP and BACE1 at the post‐transcriptional level. Furthermore, lentivirus‐mediated miR‐195 knockdown induced dementia, whereas overexpression of miR‐195 reduced dementia vulnerability triggered by two‐vessel occlusion (2VO) in rats.^85^ Similar to miR‐195, the miR‐132 level was also downregulated in the hippocampus and cerebral cortex of CCH rats.^16^, ^99^

**Table 2:** VCID‐associated microRNAs

MicroRNAs	Functions	Targets	Reference
miR‐195	Reduces dementia vulnerability and prevents VCID	APP, BACE1	Ai et al^85^
miR‐132	Protects against CCH‐induced learning and memory impairments and ameliorates dementia in VCID	Nav1.1, Nav1.2	Hu et al^16^
miR‐9	Induces cognitive impairment and promotes dementia in VCID	Nav1.1, Nav1.2 and BACE1	Sun et al^13^, Xie et al^100^
miR‐27a	Inhibits the process of autophagy and induces dementia in VCID	LAMP2	Che et al^11^
miR‐124	Inhibits the formation of Aβ and improves dementia in VCID	BACE1	Zhang et al^18^
miR‐126	Ameliorates vascular function and inhibits VCID	MMP‐9	Yu et al^101^
miR‐93	Aggravates inflammatory response and promote dementia in VCID	TLR4	Shang et al^102^, Liu et al^103^
miR‐96	Inhibits the process of autophagy and induces dementia in VCID	mTOR	Liu et al^12^
miR‐501‐3p	Aggravates BBB damage and increases the possibility of dementia in VCID	ZO‐1	Toyama et al^104^
miR‐210‐5p	Decreases synapse number and aggravates dementia in VCID	Snap25	Ren et al^105^
miR‐134‐5p	Promotes cortical neuron injury and dementia in VCID	Foxp2	Liu et al^106^
miR‐26b	Alleviates microglial inflammatory response and protects brain from dementia in VCID	IL‐6	Kang et al^107^
miR‐181c	Increases cellular adaptation to long‐term ischemia and reduces e the possibility of dementia in VCID	TRIM2,NR1	Fang et al^108^
miR‐153	Promotes synaptic plasticity damage and dysfunction and aggravates dementia in VCID	Snap25,Vamp2, Stx1a and Syt1	Zhang et al^109^, Yan et al^110^

miR‐9 is another miR involved in the pathogenesis of CCH. Sun et al discovered that the miR‐9 level was increased in the hippocampus and cerebral cortex of CCH rats after 2VO.^13^ Besides, miR‐9 knockdown reduced the symptoms of dementia triggered by 2VO in rat models.^100^ Similarly, Wei et al found that miR‐9‐5p was significantly elevated in serum and CSF in VaD patients, as well as in 2VO‐induced CCH rats. Further, the authors discovered that administration of the miR‐9‐5p antagomir significantly attenuated memory impairment, rescued the cholinergic neuronal function, and lowered oxidative stress and neuronal loss in CCH rats.^17^ CCH also enhanced the expression of miR‐27a, but inhibited miR‐124 expression.^11^, ^18^

By using endothelial cell‐specific miR‐126 conditional knockout mice, it was found that EC‐targeted deletion of miR‐126 aggravated cognitive impairment, decreased CBF, myelin density, and axon density, increased inflammation, and exacerbated water channel and glymphatic dysfunction compared with control mice in multiple microinfarction‐induced vascular dementia.^101^ Recently, several groups have demonstrated that miR‐93 aggravates inflammatory response by modulating TLR signaling pathways.^102^, ^103^

Other miRs were also reported to play a regulatory role in the pathogenesis of VaD, and modulation of these miR levels provided therapeutic potential against VCID in animal models. For example, suppression of miR‐96 expression alleviated cognitive impairment.^12^ Besides, CCH‐induced TNF‐α could upregulate miR‐501‐3p.^104^ miR‐501‐3p inhibitor effectively suppressed CCH‐induced ZO‐1 reduction and BBB destruction in cerebral white matter and significantly improved working memory deficits in the mouse model of CCH.^104^ Moreover, there was a significant increase in miR‐210‐5p in the hippocampus of rats after VCID. miR‐210‐5p antagomir effectively attenuated these VCID‐induced phenotypes.^105^ In addition, miR‐134‐5p antagomir can relieve cognitive dysfunction in VCID.^106^ On the other hand, miR‐26b overexpression significantly attenuated microglial activation, inflammatory responses, neurotoxicity, and cognitive impairments in 2VO‐generated CCH.^107^ CCH can inhibit miR‐181c expression in the hippocampus, which is closely associated with the reduction in dendrite spine density and dendritic branching of hippocampal neurons.^108^ Virus‐mediated delivery of miR‐181c partially rescued cognitive impairment in rat CCH.^108^ Direct knockdown of miR‐153 or overexpression of the antisense molecule (lenti‐AMO‐153) may be a new strategy for alleviating the synaptic pathology and cognitive decline of VaD.^109^, ^110^

### 3.3 The mechanism of microRNAs in the regulation of VCID

**Figure 2:**
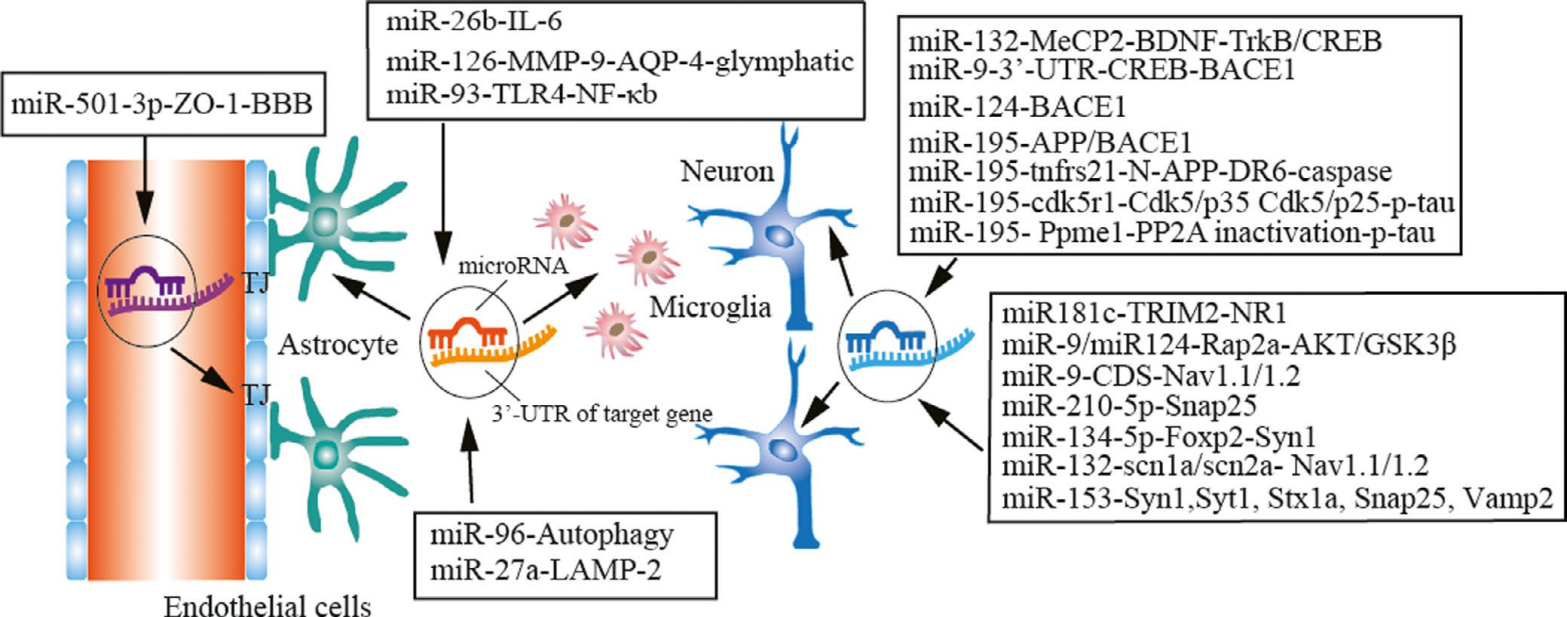
miR regulatory mechanisms in experimental VCID. miR‐132, miR‐9, and miR‐124 can regulate oxidative stress by the CREB pathway, and miR‐195 can inhibit the deposition of Aβ in neurons. miR‐181c, miR‐9, miR‐124, miR‐210‐5p, miR‐134‐5p, miR‐132, and miR‐153 can regulate synaptic loss by AKT/GSK3β and other signaling pathways in neuron dendrites. miR‐26b, miR‐126, and miR‐93 can regulate neuroinflammation. miR‐96 and miR‐27a can suppress autophagy in neurovascular units. miR‐501‐3p can decrease tight junction expression in white matter and the blood‐brain barrier

Constantly reduced blood supply and resultant neuronal hypoxia contribute to oxidative stress, which promotes neuroinflammation and BBB injury, thereby further increasing the susceptibility of the affected tissue to neurodegeneration.^111^ Recently, specific miRs have been shown to act on proteins in intrinsic and extrinsic signaling pathways through post‐translation modification, thus regulating cognitive injury after VCID (Figure 2). This section discusses the possible molecular mechanisms and signaling pathways of those miRNAs in VCID pathophysiology.

miR‐9 can target the 3’‐UTR domain of the *creb* gene to directly inhibit the expression of CREB, which suppresses BACE1 expression.^100^ miR‐9 could also regulate the process of Nav1.1/Nav1.2 trafficking in 2VO rats by binding to the coding sequence domain of Navβ2.^13^ Contrary to miR‐9, the expression of miR‐124 was continuously inhibited in the 2VO rat model. Aβ might upregulate the expression of BACE1 by inhibiting miR‐124 expression.^18^ In addition, miR‐9 and miR‐124 presented the synergistic effect in the regulation of dendritic branching by binding to the Rap GTP‐binding protein Rap2a and regulating the AKT/GSK3β pathway.^112^ By binding to the 3’‐UTR of *snap25* gene mRNA, miR‐210‐5p exacerbated cognitive impairment. The dysfunction of the miR‐210‐5p‐snap25 signaling pathway might be relevant to synaptic loss in VCID.^105^ Like miR‐210‐5p, by binding to the 3’‐UTR of *foxp2* (forkhead box p2), the miR‐134‐5p/foxp2/Syn1 pathway was found to contribute to cognitive impairment in chronic ischemia‐induced VCID through loss of cortical neurons and synaptic proteins.^106^

The reduced miR‐132 level increased tau phosphorylation at Ser396. Nimodipine alleviated cell apoptosis and reduced hyperphosphorylation of the Tau protein by activating the miR132/GSK3β pathway.^99^ miR‐132 may participate in the downregulation of methyl CpG binding protein 2 (MeCP2) after CCH, and MeCP2 downregulation was possibly involved in cognitive deficit through BDNF and its downstream pathways (TrkB and CREB) after 2VO.^14^ miR‐132 ameliorates CCH‐induced learning and memory impairments by targeting the *scn1a* and *scn2a* genes to downregulate the expression of Nav1.1 and Nav1.2.^16^

miR‐96 can suppress autophagy by regulating the PTEN‐Akt‐mTOR signaling pathway.^12^ miR‐27a affects autophagosome clearance through post‐transcriptionally regulating lysosomal‐associated membrane protein‐2 (LAMP‐2) expression.^11^ miR‐181c might improve cognitive impairment, promote hippocampal neuronal remodeling, and increase N‐methyl‐D‐aspartate receptor 1 (NR1) subunit expression through the effect of TRIM2 on neurofilament light (NF‐L) ubiquitination.^108^

miR‐195 improved dementia susceptibility in 2VO rats by inhibiting the expression of APP and BACE1 at the post‐transcriptional level via targeting different genes.^85^ In a later study, Sun et al discovered that miR‐195 could also bind to the 3’‐UTR of the *cdk5r1* gene, thereby regulating tau hyperphosphorylation. Knockdown of miR‐195 increased tau phosphorylation at Ser202/Thr205, Ser262, Thr231, and Ser422 and activated the Cdk5/p25 pathway. Overexpression of miR‐195 reversed these effects in 2VO‐induced CCH in rats.^113^ Moreover, researchers from the same group further found that miR‐195 can increase phosphatase methylesterase‐1 (PME‐1) expression by binding to the 3’‐UTR domains of the *Ppme1* gene. Downregulation of miR‐195 in CCH reversed this effect and reduced phosphatase‐2A (PP2A) protein and activity.^114^ Overexpression of miR‐195 rescued CCH‐induced dendritic degeneration and neuron apoptosis by targeting the *tnfrs21* gene to downregulate the expression of DR6 and suppress the N‐APP/DR6/caspase pathway.^15^ Considering the critical role of miR‐195 in VCID, the complement of exogenous miR‐195 may be a potentially anti‐dementia approach. A recent report showed that miR‐153 is involved in the CCH‐impaired hippocampal glutamatergic synaptic vesicle trafficking by binding site in the 3′ untranslated region (3’UTR) of the SYN1, Snap25, Vamp2, Stx1a, and Syt1 genes, which may be a new drug target for prevention or treatment of AD and VaD.^109^, ^110^

Overexpression of miR‐26b ameliorates inflammation, neurotoxicity, and cognitive impairment by decreasing the number of activated microglia and targeting IL‐6.^107^ As an angiogenic miR, miR‐126 regulates various vascular functions.^115^, ^116^ miR‐126 not only regulates angiogenesis and WM remodeling but can also regulate glymphatic function to affect innate immune response and inflammation.^117^, ^118^ Regulation of the miR‐93‐mediated TLR signal pathway is probably a potential mechanism for alleviating the inflammatory response of VCID.^119^

Given the importance of BBB integrity in VCID progression, manipulating target genes that regulate the BBB or TJ integrity may protect against VCID. Our previous data show that miR‐15a/16‐1 inhibition alleviates ischemia‐induced BBB disruption in mice.^21^, ^104^ Other miRs such as miR‐212, miR‐132, miR‐150, miR‐181a, miR‐155, miR‐501‐3p, and miR‐128‐1‐5p are also associated with the regulation of ZO‐1, occludin, and claudin‐5 expression, therefore contributing to the alterations in BBB stability.^120^ For example, TNF‐α combined with miR‐501‐3p downregulated ZO‐1 and lowered cell‐cell resistance, which plays an essential role in the pathogenesis of cerebral hypoperfusion, especially in BBB disruption.^104^

## 4 TARGETING REGULATORY MICRORNAS IS A NOVEL THERAPEUTIC APPROACH FOR VCID

miRs are key mediators in the pathogenesis of VCID. Understanding their functional significance and molecular mechanisms will provide new insights in developing novel miR‐based therapeutics to delay or rescue cognitive impairments and dementia.

### 4.1 Current methods

The current approaches for targeting miRNAs in animals include transgene or gene knockout of miRs of interest, and exogenous injection of miR mimic or antagomir/inhibitor into the vein, the lateral cerebral ventricle, parenchymal infarct area, and intranasal cavity. For instance, some researchers injected miR antagomirs into lateral cerebral ventricles or hippocampus by stereotactic technology to examine the role of specific miRNA in VCID, showing a significant neuroprotective role in the rodent experimental CCH model.^15^, ^105^, ^107^ However, intraventricular delivery of antagomir in the treatment of VCID has limitations in clinical application. A few research groups have reported that intravenous administration of specific miRNA antagomirs may be an effective therapeutic approach in experimental stroke.^21^, ^121^, ^122^, ^123^ One of the significant limitations in this field is the selection of suitable gene targeting vectors.^124^ So far, this administration method has not been reported in the clinical treatment of VCID.

### 4.2 Improving drug delivery systems for the treatment of VCID

Exosomes are vesicles (approximately 30‐100nm) derived from endosomes released from all living systems, including cells.^125^ Exosomes play an essential role in intercellular communication between the source and target cells by transferring proteomic and genomic materials, as well as proteins, mRNAs, and miRs.^125^ Comparing with routine systemic administration, exosomes, which are produced by ΒMSCs (bone marrow mesenchymal cells), due to receptor‐mediated transcytosis and benefit from their lipid bilayer encapsulation, can quickly pass through the BBB and deliver miR molecules into brain cells.^126^, ^127^, ^128^ Cell‐based exosome therapy is used to promote brain remodeling and improve neurological function. For example, after the synthesized double‐stranded miRNA was introduced into BMSCs, miR‐143 carrying exosomes were secreted and quickly transferred into osteosarcoma cells to inhibit their migration.^129^ MSC therapy has already been applied in clinical trials of stroke treatment.^130^ miR‐133b secreted by exosomes from ΒMSCs can induce neural plasticity and functional recovery in rats after stroke.^131^ Through secreting exosomes, neurons can translocate miR‐132 to endothelial cells to maintain central brain vascular integrity.^132^ Although there is no report on the treatment of VCID with various exosomal miRs, this method has broad application prospects in the future.

### 4.3 MicroRNA‐based therapy for VCID

**Figure 3:**
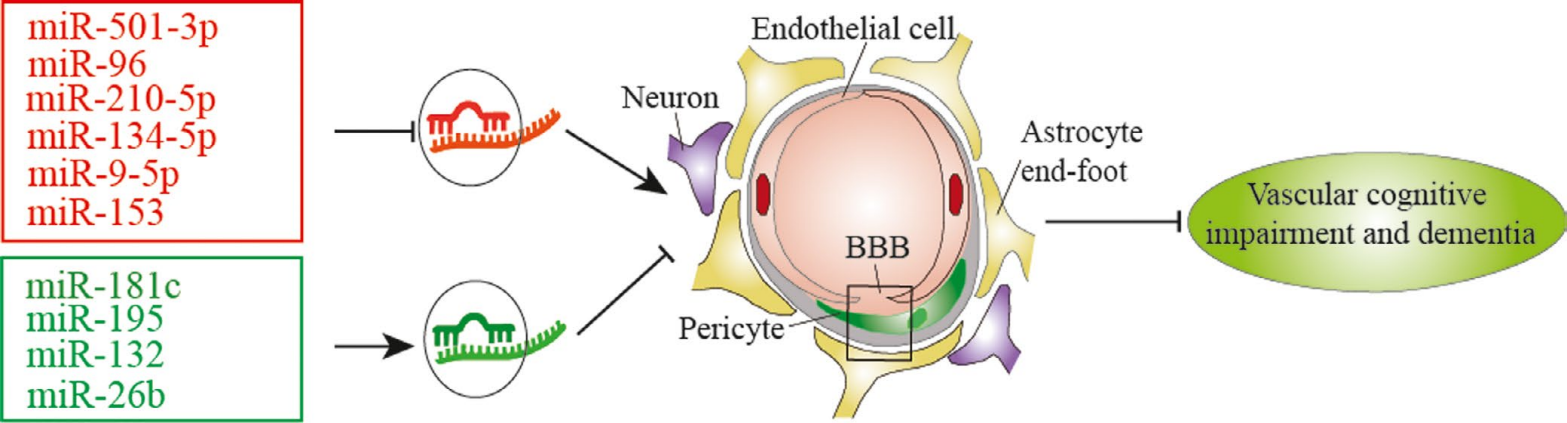
miR‐based therapy in experimental VCID. There are two therapeutic strategies to treat VCID with miR mediators: restoring downregulated miRs by using miR mimics (Green) or blocking upregulated miRs by applying their antagomirs (Red)

There are some small‐molecule chemical compounds targeting miRs to treat VCID. For example, as a candidate drug to treat tauopathy in CCH, Nimodipine has been shown to inhibit tau phosphorylation at the Ser 396 site via the miR‐132/GSK‐3β pathway.^99^ Obviously, manipulation of miRs can affect multiple signal pathways in VCID by different molecular mechanisms (Figure 3).

To improve the efficiency of miR inhibition in vivo, different chemical modifications have been developed to improve antagomir's bioavailability.^133^, ^134^ These modifications include anti‐miR oligonucleotides that are synthesized and modified by incorporations of a methyl group (2’‐O‐methyl) together with partial phosphonothioate linkage and cholesterol conjugation at the 3’ end of the strand (which improves tissue distribution and cellular uptake), and the use of locked nucleic acids (LNAs). For example, LNA‐anti‐miR‐501‐3p was intraperitoneally injected to effectively reduce BBB disruption and improve VCID in the mouse BCAS model.^104^

## 5 CONCLUSION AND PROSPECTS

miRs can translationally repress hundreds of proteins in multiple regulatory signaling pathways. Thus, physiological expression of miRs is essential for maintaining healthy development and function of the brain, whereas the imbalance of miR levels in brain cells may increase the susceptibility of VCID and other nervous system diseases. Altered miR expression and activities have been shown to play critical roles in the pathophysiology and progression of VCID.

The levels of hundreds of miRs have been found to be altered in peripheral blood samples of VCID patients, which may provide a new avenue for rapid diagnosis and treatment of VCID.^135^ Through studies from experimental animal models, it is suggested that promoting or inhibiting the expression of various VCID‐associated miRs by pharmaceutical and non‐pharmaceutical approaches is beneficial to the improvement or recovery of cognitive function in VCID.^120^ However, effective application of miR‐based therapy in VCID may encounter several challenges. A major obstacle to the effective treatment of VCID is the limited understanding of the role of miRs in its pathogenesis. At the present time, miR‐related research in VCID mainly focuses on investigating the relationship between a specific miR of interest and its regulated target genes. In the future, we need to further study miRNA regulatory networks to understand the role of their complex post‐transcriptional regulatory mechanisms. Another major challenge for miR‐based therapy in VCID is the development of pharmacological tools that can aid in effective transport of miR inhibitors and mimics across the BBB to affected brain regions with optimal concentrations.

Currently, miR‐based VCID treatment is only at the beginning stages of testing in experimental VCID models. The application of transgenic animals, especially vascular cell‐specific miR transgenic or knockout mice,^136^ can help us to better understand the mechanisms in VCID. Understanding the pathogenesis of VCID is still superficial at the present time, and subsequent research should focus on the key pathogenic mechanisms of VCID.

It is generally accepted that aging is not only a simple physiological process but also accompanied by many related complications, including hypertension, metabolic diseases, and dementia.^137^ Since VCID is closely related to aging, it is necessary for us to strengthen our understanding of the relationship between aging and its pathogenesis and clinical manifestations.^138^ There are a class of miRs that are associated with the aging process and influence lifespan by targeting components of longevity networks or by regulating stem cell behavior.^139^, ^140^, ^141^ These aging‐related miRs, including miR‐27, miR‐29, miR‐30, and miR‐71, may become the research hot spot for future miR‐based therapy in VCID.

## CONFLICT OF INTEREST

The authors declare no conflict of interest.

## Data Availability

Data sharing is not applicable to this review article as no new data were created or analyzed in this review manuscript.
